# Towards people-centred health systems: a multi-level framework for analysing primary health care governance in low- and middle-income countries

**DOI:** 10.1093/heapol/czu069

**Published:** 2014-09-11

**Authors:** Seye Abimbola, Joel Negin, Stephen Jan, Alexandra Martiniuk

**Affiliations:** ^1^National Primary Health Care Development Agency, Abuja, Nigeria, ^2^School of Public Health, University of Sydney, Australia, ^3^The George Institute for Global Health, University of Sydney, Australia and ^4^Dalla Lana School of Public Health, University of Toronto, Canada

**Keywords:** Collective governance, constitutional governance, health system actors, health system governance, low- and middle-income countries, Nigeria, operational governance, people-centred health systems, primary health care

## Abstract

Although there is evidence that non-government health system actors can individually or collectively develop practical strategies to address primary health care (PHC) challenges in the community, existing frameworks for analysing health system governance largely focus on the role of governments, and do not sufficiently account for the broad range of contribution to PHC governance. This is important because of the tendency for weak governments in low- and middle-income countries (LMICs). We present a multi-level governance framework for use as a thinking guide in analysing PHC governance in LMICs. This framework has previously been used to analyse the governance of common-pool resources such as community fisheries and irrigation systems. We apply the framework to PHC because, like common-pool resources, PHC facilities in LMICs tend to be commonly owned by the community such that individual and collective action is often required to avoid the ‘tragedy of the commons’—destruction and degradation of the resource resulting from lack of concern for its continuous supply. In the multi-level framework, PHC governance is conceptualized at three levels, depending on who influences the supply and demand of PHC services in a community and how: operational governance (individuals and providers within the local health market), collective governance (community coalitions) and constitutional governance (governments at different levels and other distant but influential actors). Using the example of PHC governance in Nigeria, we illustrate how the multi-level governance framework offers a people-centred lens on the governance of PHC in LMICs, with a focus on relations among health system actors within and between levels of governance. We demonstrate the potential impact of health system actors functioning at different levels of governance on PHC delivery, and how governance failure at one level can be assuaged by governance at another level.

**KEY MESSAGES**

- Existing health system governance frameworks focus on the role of governments in governance, and efforts to understand the governance roles of non-government health system actors in primary health care have often been limited, despite their potential capacity for governance
- Government failure in the provision of primary health care in low- and middle-income countries indicates a need for multi-level governance in which failure at one level can be assuaged at other levels of governance, including by non-government health system actors
- The multi-level governance framework presented focuses on the governance relations among different health system actors and defines three levels of governance: constitutional governance (e.g. governments), collective governance (e.g. community coalitions) and operational (e.g. supply and demand behaviour of individuals and providers within the local health market)
- Using this multi-level governance framework as a thinking guide in analyses of primary health care governance can improve our understanding, evaluation and design of people-centred primary health care systems in low- and middle-income countries

## Introduction

Health system frameworks highlight the importance of governance in explaining how health systems function and achieve desired population health outcomes. This interest in health system governance is based on the expectation that good governance leads to improved health outcomes (van Olmen *et al.* 2012). The United Nations Development Programme defines governance broadly as the exercise of political, economic and administrative authority in the management of affairs at all levels (UNDP 1997). Brinkerhoff and Bossert (2008) define governance more specifically as the rules that distribute roles and responsibility among societal actors and shape the interaction among them. However, health system governance can be difficult to conceptualize due to the challenge of accounting for the roles and relations of a broad range of actors. This becomes even more challenging given different patterns of decentralization in different settings, giving rise to multiple actors with various roles within different political, socioeconomic and cultural contexts (Barbazza and Tello 2014). Health system actors include governments, organizations, groups and individuals who, at different levels of authority, have the responsibility or capacity to carry out health system functions such as to: generate resources, deliver services, provide oversight or exert influence over decisions.

Less attention has been paid to the analysis of health system governance in low- and middle-income countries (LMICs) than to other key components of the health system (Bossert 2012). Those analyses that have been conducted have largely focused on governance as a role of governments (Bossert and Mitchell 2011). Efforts to understand the governance roles of other actors in primary health care (PHC) have often been limited, despite their potential capacity for governance (Rifkin 2009; Baatiema *et al.* 2013). This is at odds with the extensive literature on community participation and accountability in PHC which shows evidence that engaging community groups and community representatives in the provision of PHC tends to result in services that are better tailored to local needs, with better quality, uptake, accountability and health outcomes (Rosato *et al.* 2008; McCoy *et al.* 2012; Molyneux *et al.* 2012). It is also at odds with the recognition that when government regulation of health systems is weak, PHC governance may become by default the responsibility of health workers and the local health market (Bloom *et al.* 2008, 2011). In addition, people in communities do seek out means of assuaging inadequate government support. They do this for example by raising funds to support PHC, monitoring PHC facilities and health workers and advocating to governments for improved support (Rosato *et al.* 2008).

Health systems may be regarded as people centred when the potential roles and capabilities, and the needs and preferences of individual actors (e.g. service users, health workers and health managers) and collective actors (e.g. whole communities and community groups) are recognized and given priority in the day-to-day operations of the health system. To achieve people-centred health systems, there is a need for an approach to governance which incorporates the roles and relations of all health system actors. However, existing frameworks, and the growing literature on health system governance, focus mainly on the role of governments in governance. In a synthesis of this literature, Barbazza and Tello (2014) identified three emerging areas of focus: (1) characterizing the concerns of governance (e.g. provision of public goods, ethics and integrity, control of corruption and rule of law); (2) operationalizing the functions of governance (e.g. accountability, policy direction, regulation and participation); and (3) specifying the outcomes of governance (e.g. efficiency, equity, risk protection and quality). Examples of health system governance frameworks include Siddiqi *et al.* (2009), Baez-Camargo and Jacobs (2011), Mikkelsen-Lopez *et al.* (2011) and Fattore and Tediosi (2013). While they can be used to explore the performance of governments in health system governance, these frameworks do not emphasize the role of non-government health system actors in governance.

In addition to governments, health system governance frameworks by Brinkerhoff and Bossert (2008) and Cleary *et al.* (2013), also focus on non-government health system actors such as health workers and service users as important health system actors. Based on a World Bank (2004) framework of accountability relationships in service delivery, Brinkerhoff and Bossert (2008) frame the relations among three categories of health system actors (governments, providers and service users) as governance relationships which ultimately affect health service delivery. Using the framework put forth by Brinkerhoff and Bossert (2008), Cleary *et al.* (2013) explored accountability relations as a governance function among the three categories health system actors at the PHC level in LMICs. For instance, Cleary *et al.* (2013) show that a focus on compliance to outputs and targets defined by a strong central government (bureaucratic accountability) can constrain the efforts of frontline providers in responding to the needs of service users (external accountability). Cleary *et al.* (2013) also show that the resources available to different health system actors, their attitude, and organizational culture influence the relations among health system actors at the PHC level in LMICs.

In this article, we propose a multi-level governance framework for use in analysing PHC governance. While focusing on a similar range of actors (governments, providers and patients/citizens) as Brinkerhoff and Bossert (2008) and Cleary *et al.*(2013), this framework differs by situating them as not only actors, but also as potential governance practitioners (for ‘good’ or ‘bad’) within the health system. Unlike previous health system governance frameworks, the multi-level governance framework also differs in its focus on how actions are conducted and decisions are influenced within the health system. The multi-level framing of PHC governance is at three levels: one level about the individual actions and decisions of health system actors (operational governance), another level about the collective actions and decisions of health system actors (collective governance), and the third level about the actions and decisions of governments. This potentially allows for a more detailed exploration of governance arrangements within PHC systems in LMICs. Beyond the mere recognition of people as important health system actors, the multi-level governance framework situates the activities of non-government health system actors within defined levels of governance, given that they often perform governance functions, especially at the PHC level in LMICs.

This article describes the multi-level governance framework and its application to PHC. Using the example of PHC governance in Nigeria, we illustrate how the framework may work in LMICs as a people-centred thinking guide to link the roles of different health system actors to PHC delivery.

## The Multi-Level Framework for PHC Governance

The multi-level framework is a component of the larger Institutional Analysis and Development framework (Ostrom *et al.* 1994). The multi-level governance framework is used extensively in the social sciences to analyse the governance of common-pool resources (Pottette *et al.* 2010; Ostrom 2010). The peculiarity of common-pool resources is that they are owned by everyone in a community, and not anyone in particular, potentially leading to overuse and degradation, a situation termed the ‘tragedy of the commons’ (Hardin 1968). Examples include open access parks, groundwater basins, irrigation systems, lakes, fisheries and forests. The extensive body of research (see Pottette *et al.* 2010) that resulted in the multi-level governance framework showed that depending on the context, individuals and communities in common-pool resource settings do take on governance roles on their own. The ‘tragedy of the commons’ is not inevitable. People can evolve coalitions of resource users with rules of self governance to avoid overuse and degradation. This finding contradicts earlier claims (Hardin 1968) that common-pool resources must be regulated by the government or be privatized. It supports the evidence that individuals and communities can assuage the effects of government failure in the provision of public goods such as PHC in LMICs (Lewis 2006; Akinola 2007). This is important because, to the extent that they are commonly owned by a community, PHC services in many LMICs are similar to common-pool resources (Ostrom 1996).

### Common-Pool Resources and PHC

Part of the research that led to the multi-level governance framework (Ostrom *et al.* 1994; Polski and Ostrom 1999) was conducted in common-pool resource settings such as community owned irrigation systems. With a finite quantity of resource units, individuals make day-to-day decisions on the extent to which they will seek to maximize benefits from the irrigation system. This is because each person’s use of the irrigation system subtracts from the state of the infrastructure and the overall quantity of water available to other people in the community. The decisions of individuals, who may seek to maximize the benefits of the irrigation system for their own farmland, are shaped by (1) individual choices; (2) the existence of collective arrangements among farmers in the community on how to use or not use the irrigation system; and (3) the extent to which individuals comply with these agreements. When an irrigation system is owned by a government, a non-governmental organization (NGO), or is privately provided, the behaviour of people using it may also depend on whether or not there are rules or policies made by the providers, and the extent to which they are enforced (Ostrom 2010). The rules and arrangements are further conditioned by the physical, socio-economic, cultural and political context in a community (Ostrom 2010).

This range of influences on the management of common-pool resources also applies to PHC services in a community. Similar individual and collective actions and decisions in the community are important to ensure the supply of PHC services and the maintenance of PHC facilities in LMICs, especially where government support for and regulation of PHC may be limited (Das Gupta *et al.* 2003; Rosato *et al.* 2008). The decision of non-government actors to support or not support PHC may be shaped by individual interest in maximizing the benefits of having a PHC facility in the community. Individuals may invest in the PHC facility or make direct contributions to the PHC facility to ensure its operations. They may also be motivated by collective arrangements in the community to provide support for PHC, through community insurance schemes or financing initiatives to support the PHC facility. However, the decision of local non-government health system actors to support PHC in a community may depend on whether government policies allow for such individual contributions or investments, and collective action at the community level.

Initiatives of health system actors other than governments can contribute to ensuring the availability of PHC services. For example, a 2003 survey of PHC facilities in Kogi State, Nigeria showed that community health committees were the main source of support for building maintenance in 57% of 140 PHC facilities (Das Gupta *et al.* 2003). Health workers were also the main source of drugs and medical supplies in about 15% of the PHC facilities. This study showed that the longer staff were unpaid, the more likely it was that essential drugs were provided by facility staff, such that earning informally from drug sales became a strategy to augment their irregular income (Das Gupta *et al.* 2003). A study of similar ‘informal’ economic activities among PHC workers in Uganda (McPake *et al.* 1999) also showed that levying informal charges for services was associated with lower levels of health worker absenteeism and higher levels of service utilization. These informal economic activities may reflect the level of demand for health services: a community in which demand for PHC services is high may experience such informal activities to support the continued running of the PHC system, where formal mechanisms do not exist or break down.

Like common-pool resources, individual and collective actions and decisions of non-government actors is often a component of the governance of PHC in LMICs. Like community fisheries or irrigation systems in previous applications, the multi-level governance framework is also applicable to PHC in a community. In previous applications of the multi-level governance framework, the goal of analysis was to understand how to preserve common-pool resources, while in this application, the goal of analysis is to understand how to ensure not only the optimal supply of, but also the demand for PHC services. The roles of community groups in preserving common-pool resources featured prominently in previous applications of the framework. In this application, we also highlight the role of community groups in ensuring optimal supply and demand of PHC services as shown in previous work on community participation in PHC (Rosato *et al.* 2008). Previous applications of the multi-level governance framework showed how the activities of and the relations between governments, community coalitions and day-to-day behaviour of individuals in a community affect the depletion of a common-pool resource unit. This is also the case in this application, but with a focus on PHC services.

### The Multi-Level Framework: Operational, Collective and Constitutional Governance

**Figure 1:**
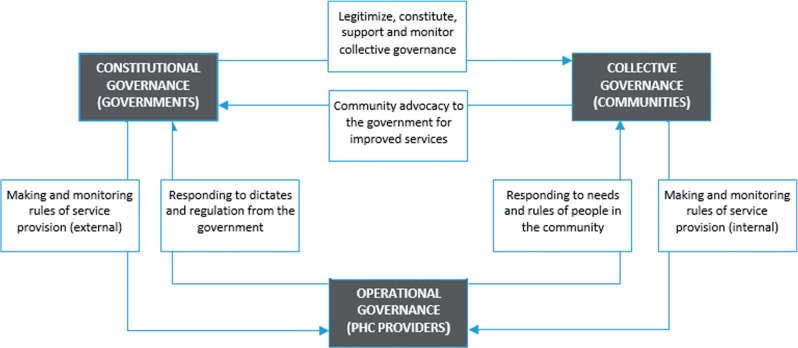
The multi-level framework for PHC governance in LMICs. Note: The actors that occupy each level of governance vary with the policy issue or objective of analysis. In this rendering of the framework, PHC providers are at the operational level, communities are at the collective level and governments are at the constitutional level. Other renderings of the multi-level framework may have or include individual service users at the operational level, specific community groups at the collective level or large NGOs and similar organizations at the constitutional level.

The components of the framework consist of three levels of PHC governance. Figure 1 shows a simplified version of a complex set of interactions among the three levels of governance. These levels are defined as operational governance, collective governance and constitutional governance:

1. **Operational governance** is the process by which individual local health system actors make decisions on the demand and supply of PHC services in their community. Operational governance refers to how individuals and health providers in the local health market make and implement practical decisions on day-to-day activities based on individual choices and market forces, or as allowed by collective governance in the community and constitutional governance by governments (McGinnis 2011).
2. **Collective governance** refers to collective action by community groups or representatives who bring communities into partnership with their PHC providers. The group may be a coalition of PHC facility users who came together on their own to support its operations. The group may also be constituted as an intervention to stimulate support for PHC. The group may include appointees of community leaders or the government. These community groups may (internally) set, change, influence, monitor and enforce the rules guiding the demand and supply decisions of individual health system actors in the community. The actions and decisions of community groups, however, depend on whether or not they are authorized (or allowed) by constitutional governance processes (McGinnis 2011).
3. **Constitutional governance** refers to the actions and decisions of governments and similar bodies in setting, dictating and influencing the rules governing collective and operational actions and decisions. It is the level at which entities involved in collective and operational processes are defined and legitimized. Health system actors at the constitutional level (externally) make, change, influence, monitor and enforce rules as contained in a constitution or policy documents (McGinnis 2011). While typically distant from the field of operation, constitutional governance could obtain at different levels, such as different tiers of government, and may arise from non-government actors such as traditional leaders, religious leaders, national and international NGOs and global health organizations.

A health system actor may belong in different categories, depending on the actions they take and the decisions they influence. People acting alone belong at the operational level, whereas an individual acting as part of a group functions at the collective level of governance. A small NGO within a community may act at the collective level of governance in relating to a public PHC facility. A larger NGO with widespread national or international presence may act at the constitutional level of governance. When a faith-based organization within a local community owns and runs a non-profit PHC facility, the faith-based organization functions at both collective and operational levels of governance. A for-profit provider functions at the operational level, and its board of governors, if selected from the community may function at the collective level of governance. However, irrespective of the arrangements in a community, without effective governments, actors at either the collective or operational levels of governance take on roles of constitutional governance (Bushouse 2011). On the other hand, excessive exercise of constitutional governance in regulation, performance management or accountability demands can crowd out the responsiveness of health system actors at the operational level to patients and citizens functioning at either the operational or collective level of governance (Cleary *et al.* 2013).

The approach to using this multi-level governance framework is based on the premise that the optimal delivery of PHC services depends on the strength and nature of relations among all health system actors, with each performing the roles for which their well suited, based on their capability to make, change, monitor and enforce rules governing demand and supply of PHC services (Ostrom 2010; Bushouse 2011). The rules that guide these relations may be formal ‘rules-in-form’ (laws, policies and regulations) or informal ‘rules-in-use’ (social norms, convention and shared strategies) (Ostrom 2010). In the discussions that follow, we apply this multi-level framework to the governance of PHC in Nigeria, illustrating how the framework may be applied to PHC governance in LMICs generally.

## The Multi-Level Governance of PHC in Nigeria

The governance of PHC in Nigeria reflects the relations between local health markets, communities, sub-national governments, the national government and global agencies, with overlapping centres of authority and responsibility, which interact to determine the rules under which people can act in the demand and supply of services (McGinnis 2011). In this section, we discuss the application of the multi-level governance framework to the PHC system in Nigeria, its implication for the design of PHC governance in Nigeria, and potential limitations of applying the framework to PHC governance.

### Operational Governance of PHC in Nigeria

In Nigeria, communities have tacit rules about health care-seeking behaviour, such as what people with symptoms of tuberculosis are expected to do, how women are expected to seek care during pregnancy and childbirth, and the need for childhood vaccination. Using these rules, individuals in a community make operational choices when they have symptoms of tuberculosis, are pregnant or in labour or wish to vaccinate a child. Their choices depend on personal preferences, shaped by community norms and the local health market. These also exist within and are subject to broader influences of constitutional governance. In addition, providers (public, non-profit or for-profit) within a local health market can set up rules for one another on how to co-ordinate and provide incentives for referrals in order to reduce patient delays. Health workers can also have tacit rules among themselves about profiteering or absenteeism. The same applies to the choices health care providers make in service delivery and addressing needs in a community: e.g. whether to conduct community outreach to improve vaccination coverage, or provide home deliveries to increase skilled birth attendance or perhaps, whether to demand illegitimate charges for services or exempt the poor people in the community from service charges. These actions and decisions may in turn influence community demand for services, and may also be influenced by collective rules in the community. The actions and decisions of health providers in a community also exist within and are subject to broader influences of constitutional governance.

### Collective Governance of PHC in Nigeria

In Nigeria, the prescribed model of collective governance of PHC is such that each community has a PHC committee (Oyegbite 1990; FMOH 2004), the membership of which includes the primary school head teacher, the health worker in charge of the PHC facility, representatives of the town union and traditional, voluntary, religious, women, youth and health-related occupational groups, such as traditional healers, birth attendants and patent medicine vendors. The chairman is elected by the members of the committee, which are expected to meet at least once a month (Uzochukwu *et al.* 2004). Although the government shapes the structure of these committees, and sometimes initiates them, their activities are not subsidized by the government. The committees are typically formed by a participatory approach to assist communities in identifying unsatisfied demand and finding appropriate solutions. Their roles include influencing demand-side operational rules through community health education and their decisions about community health outreach. They also influence supply-side operational rules by deciding on activities in the PHC facility, supervising traditional birth attendants and community health workers and monitoring the performance of the PHC facility (NPHCDA 2012). However, any group within the community, whether or not they are primarily constituted for reasons related to PHC, may be involved in the collective governance of PHC. For example, an ethnic minority in a community or a faith-based group may have collective rules on PHC or may establish their own non-profit or for-profit PHC facility. Likewise, a women’s group may have specific arrangements with PHC providers for the care of children and pregnant women.

### Constitutional Governance of PHC in Nigeria

The federal system of government in Nigeria functions at three tiers: national, state (provincial) and local (district) governments. In the Nigerian constitution (FRN 1999), PHC governance is decentralized as a joint responsibility of the sub-national governments i.e. states and local governments. However, much of PHC provision is left to local governments, where the financial and technical capacity to deliver PHC is least available (Oyegbite 1990; Okorafor 2010). This is because the decision on which of them takes primary responsibility for PHC depends on the constitutional choice of each state government and state governments are free to determine the extent of support they provide for PHC. The national government provides overall policy direction for PHC, advocates to states and local governments to improve support PHC and supports them where and when necessary in order to achieve national and global health goals (Sorungbe 1990). In addition, governments in Nigeria are influenced by global health actors and aspire to meet global health goals and targets (see Appendix 1).

Local governments in Nigeria are particularly weak because of the funding arrangements among the tiers of government. Although tax revenues are raised by the national and sub-national governments, the majority of revenue from tax and resources are owned by the national government (Olakunde 2012). Revenue generated by the national government is remitted to the Federation account. Most sub-national governments depend entirely on allocation from the Federation account because of their low capacity for internally generated tax revenue, given that the majority of Nigerians are poor (Olakunde 2012). Revenue generated by the national government is shared among governments according to a formula that keeps about half of the funds at the national level, a quarter for the 36 states, and the other quarter for the 774 local governments (Lukpata 2013). However, there are no rules by which sub-national governments can earmark these funds according to sector (Oyegbite 1990) and there are also no rules requiring sub-national governments to provide budget and expenditure reports to the national government (Olakunde 2012). In line with the constitution, funds allocated to local governments are channelled through accounts held by state governments. Without rules prohibiting states from withholding local government funds, state governments decide how much of local government funds reach them (Okafor 2010), hence the weakness of local governments in Nigeria, and of their role in the constitutional governance of PHC.

### Polycentric Governance of PHC in Nigeria

The pattern of decentralization of PHC governance in Nigeria results in an unpredictable and uneven PHC system across Nigeria. Health outcomes depend on how local political, cultural, socioeconomic and physical circumstances influence the supply and demand of PHC services within the local health market (Babalola and Fatusi, 2009; Ononokpono and Odimegwu, 2014). These community circumstances shape responses at the operational or collective levels of governance to potential failures at the constitutional level of governance. Actors at the constitutional level of governance may also respond to weaknesses at other levels of governance. In the absence of constitutional and collective governance, PHC services are provided according to decisions of PHC workers and other individual actors in the local health market (i.e. operational governance) (Bushouse 2011). This way, actions and decisions at one level of governance can assuage the effects of weaknesses at other levels of governance.

Multi-level governance systems such as the PHC system in Nigeria are described as ‘polycentric’ when the different levels of decision making are formally independent of each other, whether or not they actually function independently (Ostrom *et al.* 1961). But in Nigeria, the extent to which each level of PHC governance can possibly be independent of the other is limited, given that lower levels of governance are nested into higher levels. For example, PHC facilities and communities operate within the frame of local governments, which in turn operate within the frame of state governments. Furthermore, state governments operate within the frame of the national government. However, empirical research on the governance of common-pool resources and public goods (McGinnis 1999) show that in spite of potential redundancies, polycentric governance systems tend to achieve higher levels of performance than single-unit, central authority governance systems. This is especially the case when polycentric governance allows for proximity of governance to the people, tailored to the specific circumstances of each community, and for checks and balances, small-scale learning and manipulations, multi-level discussions, competition of ideas and backup in case of failures at other levels (Brondizio *et al.* 2009).

### (Re-)designing PHC Governance in Nigeria

The multi-level governance framework has practical implications for the design of PHC governance (see Appendix 1 and 2 for further examples of governance interventions). We illustrate this by discussing the strategies used by the Nigerian government to address dysfunctions in PHC governance in Nigeria. The national government of Nigeria first responded to the disjunction between capacity and responsibility in the constitutional governance of PHC when, in 1992, it established the National PHC Development Agency (FRN 1992). Through the National PHC Development Agency, the national government provides policy, strategy, oversight and technical support, while states and local governments provide logistics and human resources to implement services. However, as the National PHC Development Agency is an agency of the national government, it cannot impose programmes or policies in states and local governments.

**Figure 2:**
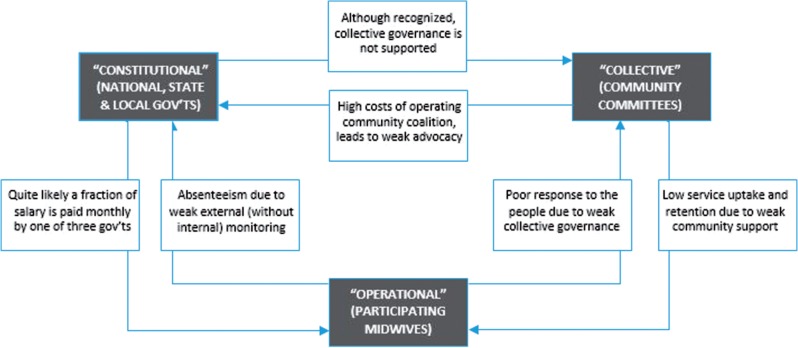
Application of the multi-level governance framework to the Midwives Service Scheme in Nigeria shows a failure of polycentric governance due to weak collective governance

The Midwives Service Scheme in Nigeria illustrates the limits of the National PHC Development Agency. In order to expedite Nigeria’s achievement of the Millennium Development Goals, the national government in 2009 addressed a key driver of high maternal and child mortality in Nigeria: the failure of sub-national governments to provide human resources for skilled birth attendance (Abimbola *et al.* 2012). The Midwives Service Scheme was established as a collaborative effort among the three tiers of government, with the National PHC Development Agency representing the national government. The Midwives Service Scheme aims to reduce inequities in access to skilled birth attendance by redistributing midwives from urban to rural areas; however, persisting governance challenges limit the success of the scheme. These include varying levels of commitment by state and local governments across the country, such as failure by some local governments to keep a commitment to provide free accommodation to midwives. In addition many states and local governments fail to keep their commitment to share payment of the midwives among national, state and local governments in a ratio of 3:2:1 and to effectively monitor and supervise the midwives within their jurisdiction (Abimbola *et al.* 2012). Absenteeism and retention of midwives in the scheme is a major challenge. The National PHC Development Agency often relies on ineffective strategies to co-opt states and local governments to fulfil their statutory roles. Also, despite the availability of skilled birth attendants in participating communities, women are still more likely to deliver at home without skilled attendance (Abimbola *et al.* 2012), due in part to weak collective governance (Figure 2).

Since 2010, reform efforts of the national government have included advocacy to bring ‘PHC under one roof’ of state governments by creating a state-level agency that is empowered to take responsibility for PHC. The state agency will be more accountable to the national government through a constitutionally sanctioned shared funding platform, in which each state has its own replica of the National PHC Development Agency, called the state PHC board (NPHCDA 2013). However, while the national health policy and the ‘PHC under one roof’ policy recognize collective governance, and although implementation of Midwives Service Scheme includes establishing a PHC committee in each participating community, there is no commitment to provide ongoing technical and financial support for collective governance. Establishing formal support for collective governance, beyond mere recognition, may strengthen PHC governance within the Midwives Service Scheme (Golooba-Mutebi 2005). Such support for collective governance is particularly important in settings where the failure of constitutional governance can be taken for granted (Lewis 2006), else the governance of PHC will be left to the actions and decisions of PHC workers and other individual actors within the local health market (i.e. operational governance) (Bushouse 2011).

The polycentric system of governance in which the national, state and local governments, the community and the local health market are responsible for PHC has the benefit of ensuring that if one tier of government fails, another can step in to ensure that PHC services are available in a community. Although the policy to bring ‘PHC under one roof’ may streamline governance, reduce redundancy and create accountability, there is a risk of reducing polycentricity. The policy increases the likelihood that if a state government fails, PHC might fail in the entire state. In addition, by reassigning the primary responsibility for PHC to state governments instead of local governments which are closer to the people, the policy further distances constitutional governance from the people. This increases the likelihood of failure of community advocacy or lobbying from the collective and operational levels of governance. The increased distance from the community also reduces the capacity to monitor and enforce constitutional rules (Bushouse 2011). However, while the ‘recentralization’ policy of ‘PHC under one roof’ can reduce polycentricity, community coalitions, being an additional layer of governance, can increase polycentricity. Therefore, it would be appropriate to match the ‘recentralization’ of PHC governance with active support for collective governance to make the system more robust to potential government failure.

## Limitations of the Multi-Level Governance Framework

In using the multi-level governance framework as a thinking guide, it is important to be mindful of several factors that may limit its application to PHC in LMICs. Firstly, non-government actors tend to require external support in their response to unsatisfied demand due to relatively higher costs of providing PHC. This includes the cost of construction and equipment, of drugs and medical supplies, and the cost of staffing a PHC facility (Golooba-Mutebi 2005). Second, non-government actors may also require external support because of the information asymmetry that characterize health care markets (Arrow 1963). Information asymmetry implies that the additional knowledge possessed by health providers places them in a position of power and advantage in health care transactions. Thus external support may be required in obtaining information about unsatisfied demand and in facilitating responses to those unsatisfied demands for PHC (Björkman and Svensson 2009). Third, external intervention may be necessary when individual or collective actions and decisions of non-government health system actors systematically favour (e.g. high-income members) or exclude (e.g. ethnic minority members) certain members of the community (McCoy *et al.* 2012).

In addition, a polycentric system of governance in which each level of governance is truly independent of the other is neither feasible nor desirable in PHC governance. The literature on community accountability indicates that in many LMIC settings, national and sub-national government support to communities may be necessary to reduce information asymmetry and to ensure effective community accountability at the PHC level (Björkman and Svensson 2009; Molyneux *et al.* 2012). The power relations in the health system also necessitates the regulatory role of governments (Bloom *et al.* 2008), reflecting in turn that actors at the constitutional level of governance can exercise power over actors at the collective and operational levels of governance (Erasmus and Gilson 2008). However, polycentricity in PHC governance may be enhanced in some LMICs where failure of constitutional governance to provide or regulate the provision of PHC services (Lewis 2006), leaves much responsibility and power at other levels of governance.

While the framework draws attention to particular features of PHC systems in LMICs, it is not sufficient to analyse specific governance functions. The analysis of specific functions, such as transparency and accountability require specific frameworks designed for that purpose such as: Brinkerhoff and Bossert (2008); Siddiqi *et al.* (2009); Baez-Camargo and Jacobs (2011); Mikkelsen-Lopez *et al.* (2011); and Cleary *et al.* (2013). However, there is a need for further analytical frameworks to inform efforts towards making health systems more people centred. Further research should also explore how context influences the capacity of government and non-government actors to perform health system governance roles. In a review of the literature on accountability mechanisms at the PHC level in LMICs, Cleary *et al.* (2013) framed such contextual factors as: (1) the resources (time, space and capacity) available to health system actors, (2) their attitudes and perceptions on their roles in health system governance and (3) the values, beliefs and culture which shape the actions and decisions of the health system actors. This framing of contextual factors influencing governance may be used as a starting point for comparative case studies to further inform the understanding of PHC governance in LMICs.

## Conclusions

In summary, we have described a multi-level governance framework, which has previously been used to analyse the governance of common-pool resources. We have also proposed and demonstrated its use in analysing PHC governance in LMICs. This is because of the similarities between common-pool resources and PHC in LMICs. These similarities include a tendency to be commonly owned by the community and no one in particular, leading to the need for individual and collective action to avoid the ‘tragedy of the commons’. The multi-level governance framework offers a people-centred lens on the governance of PHC in LMICs, with a focus on the dynamic relations among health system actors, the nature of their actions and choices (individual, collective or constitutional), how their actions and decisions may contribute to the governance of PHC at different levels, and their potential impact on PHC delivery in a community.

Application of the framework to PHC governance in Nigeria (see also Appendix 1 and 2) led to some ideas for analysis and action for strengthening PHC systems in LMICs. For example, health policy and system researchers should:

- Include the relations among a broad range of actors in the health system (including governments and non-government actors) in analyses aimed at troubleshooting and reforming PHC governance systems.
- Investigate in different settings how variations in governance arrangements and relations among different levels of PHC governance can affect service delivery and outcomes.

In addition, efforts to improve PHC governance by policy makers and health systems practitioners should:

- Avoid concentrating authority and responsibility at one level, but instead promote governance at more than one level while aiming for clear lines of authority and accountability.
- Recognize and support the roles and potential of non-government health system actors in responding to unsatisfied demand for PHC services in communities.

There is no ideal type of multi-level governance, given that individuals and communities in different LMIC settings have varying resources, attitudes and cultures which influence PHC governance. Applying this multi-level governance framework to PHC in different LMIC settings will therefore depend on the local context, including existing political models. However, we hypothesize that using this framework will improve our understanding, analysis and design of more people-centred PHC systems in LMICs.

## Appendix 1

### Linking Governance and Service Delivery: Polio Eradication in Nigeria

The global goal to eradicate polio encountered a setback when in 2003–04 the rejection of polio vaccination in northern Nigeria followed rumours that the vaccine contained chemicals that would sterilize children and reports that an unregistered drug was used in northern Nigeria during a meningitis outbreak in 1996 which left several children paralysed (Jegede 2007). The strategy used by national and global health leaders to change rejection of the polio vaccine was to engage religious and traditional leaders in northern Nigeria. In particular, the Sultan of Sokoto, the pre-eminent Muslim leader in northern Nigeria, was engaged to help reverse the trend of polio vaccines refusals given his strong influence on the predominantly Muslim population in the affected communities in northern Nigeria. Muslim clerics were enlisted to preach to their congregants messages on the need to accept the polio vaccine. Traditional rulers in northern Nigeria also supported the polio eradication initiative as a result of the influence of the Sultan of Sokoto. In addition to the religious and traditional rulers, political leaders at the national and sub-national levels also added their voice to a call for people in the affected communities to accept the polio vaccine (Abimbola *et al.* 2013).

Engaging these actors at the constitutional level, that is traditional and religious leaders, helped to improve acceptance of the vaccine. This is because, for some people in some communities, constitutional rules made by traditional and religious leaders became rules-in-use at the operational level. However, the success of engaging constitutional governance to resolve the rejection of polio vaccination was limited partly because of the large jurisdiction over which constitutional governance superintends and because of its distance from communities. The capacity of distant constitutional actors to effectively communicate and change, monitor and enforce rules is limited (Cooke and Tahir 2012). Their role in monitoring and enforcing rules is *external*, in contrast to the more effective *internal* monitoring and enforcement of rules possible with health workers and a community coalition that is responsible for only one health facility and a community (Bushouse 2011). One PHC facility is usually a minor responsibility of constitutional governance. Thus, monitoring and enforcing rules governing the demand and supply of its services is costly and may be beyond the immediate concern of constitutional governance (Bushouse 2011).

Without collective governance, the monitoring and enforcement of rules is weak, communication is ineffective (Renne 2006; Nasiru *et al.* 2012), and the responsibility to communicate, build trust and change rules falls on health workers at the operational level who may not respond to the needs and preferences of people in the community (Bushouse 2011). Success depends on whether the responsibility to communicate, build trust and change rules rests exclusively at the constitutional level or is shared with health system actors at the operational level and members of the community with whom people can share collective rules. For instance, a study in northern Nigeria (Mangal *et al.* 2014) showed that while obtaining health information from key religious leaders strongly affected the probability that a child had received the polio vaccine, information provided by health workers and community representatives (town announcers) had a more significant effect on the probability that a child is vaccinated against polio.

### Appendix 2

#### Considering Interventions at Different Levels of Governance

In rural communities in southern Nigeria, a study of the costs of tuberculosis (TB) care showed that 45% of total costs were incurred by patients before diagnosis (Ukwaja et al. 2013a). These costs, representing the transaction costs of access to care (Stiles et al. 2001), were incurred before reaching a health facility where the disease could be diagnosed. This is due to lack of good rural road networks, affordable transport and communication services, and also due to information asymmetry, which lead to patients seeking and obtaining inappropriate care from informal providers such as drug stores and traditional healers who operate within a weak regulatory framework with ineffective monitoring and enforcement of rules (Ukwaja et al. 2013b). For the rural poor, seeking care from informal health providers is often a strategy devised to cut costs, but by which they may end up incurring higher costs due to opportunism in the poorly regulated rural health market (Bloom *et al.* 2011). Efforts to reduce the transaction costs of access to TB care in a community may be considered at one or more levels of governance as follows:

- At the operational level, formal TB service providers may give incentives to informal providers to ensure early and immediate referral when patients present with symptoms suggestive of TB. However, the costs incurred in initiating this arrangement include the costs of negotiating and enforcing agreements between formal and informal providers within the local health market. This cost may be high, given the financial interests of informal providers in benefiting from high transaction costs of access to care (Oladepo and Lucas 2013).
- At the constitutional level, the government may enforce regulations that limit the activities of informal providers. Drug stores for example may only be able to dispense over the counter drugs. The costs of administering this intervention may be high due to the difficulty of distant government agents to effectively monitor and enforce rules in a community (Bloom *et al.* 2011). This is especially so, given that the rules will prohibit trusted health providers or services, which have been around much longer, are more readily available, less costly and have closer relations with the community than formal providers (Breiger et al. 2004; Abimbola 2011). The costs of administering interventions beyond the health system to reduce transaction costs may also be high, e.g. free education for the rural poor, building better rural road networks, and funding low cost transportation and communication (Altmann 2011).
- At the collective level, a community group may address information asymmetry by disseminating information about the appropriate formal provider of TB services. While interventions at the collective level may be less costly than at the operational and constitutional levels due to proximity, nevertheless, the upfront costs of administering the intervention (such as costs of travel to hold public consultations and meetings) may still be high enough to constitute an obstacle to collective action (Varughese and Ostrom, 2001). Given this potential obstacle, a community coalition may collaborate with formal providers (operational governance) and the government (constitutional governance) to promote, and enforce cost-reducing referral practices among informal providers.
